# Role of Monoclonal Antibody "Alemtuzumab" in the Treatment of Multiple Sclerosis

**DOI:** 10.7759/cureus.13246

**Published:** 2021-02-09

**Authors:** Sadia Nosher, Sehrish Fuad, Nupur Mishra, Zaid A Alrashid, Bindu Rathod, Devyani Mohan, Deepak M Basavanagowda, Arveen Kaur, Stacey E Heindl

**Affiliations:** 1 Internal Medicine, California Institute of Behavioral Neurosciences & Psychology, Fairfield, USA; 2 Medicine, California Institute of Behavioral Neurosciences & Psychology, Fairfield, USA; 3 Neurology, California Institute of Behavioral Neurosciences & Psychology, Fairfield, USA; 4 School of Medicine, Spartan Health Sciences University, Vieux Fort, LCA; 5 Psychiatry and Behavioral Sciences, California Institute of Behavioral Neurosciences & Psychology, Fairfield, USA; 6 Surgery, California Institute of Behavioral Neurosciences & Psychology, Fairfield, USA; 7 Medicine, Avalon University School of Medicine, Willemstad, CUW

**Keywords:** immune-mediated thrombocytopenia, multiple sclerosis, alemtuzumab, secondary autoimmune diseases, disease-modifying therapies, nephropathy, thyroid disorders

## Abstract

This article will review current treatment options for multiple sclerosis (MS) while keeping our primary focus on alemtuzumab, as it is now approved in more than 65 countries. From a pathophysiological point of view, MS is a disabling disease impacting a patient's life both physically and mentally, leading to devastating social and economic impact. This review will elaborate on alemtuzumab's role in treating relapsing-remitting MS (RRMS) by comparing its efficacy, side effects, and monitoring with other disease-modifying therapies (DMTs) available in the market. It is a point of great concern not only for physicians but also for neurologists, nephrologists, endocrinologists, dermatologists, and oncologists when encountering long-term effects of alemtuzumab in the life of treated MS patients. We hope that our review will not only benefit treating faculties but also those who are suffering from this devastating disease.

## Introduction and background

Multiple sclerosis (MS) is a chronic, inflammatory, neurodegenerative autoimmune disease caused by proliferation and activation of autoreactive lymphocytes that react against unidentified autoantigens and start inflammation in coordination with proinflammatory cytokines [1]. Regulatory B cells are immature transitional B cells (CD19+, CD24, and CD38) that tend to have regulatory action through the production of IL-10 [2]. Another study showed that B cells carry programed death ligand (CD19+PD-L1hi cells) that produces its regulatory effects through cell-to-cell contact through the interaction of (CD19+PD-L1hi cells) with PD-1 on T cells, which results in the termination of T follicular helper (Tfh) cell differentiation and proliferation, leading to the relapse of symptoms of MS patients [3,4]. Alemtuzumab depletes B and T cells through CD52 ligand, which is present in high amounts on these cells than on natural killer cells and other immune cells, which are part of innate immune cells leading to less inflammatory effects of circulating B and T cells [5,6]. After three to 12 months of treatment with alemtuzumab, regulatory B and T cells, and memory B and T cells will repopulate and rebalance the immune system [7]. According to the hypothesis, the reconstitution of B and T cells may result in the hyperpopulation of immature B cells resulting in other autoimmune diseases such as Graves' disease, immune-mediated thrombocytopenia (ITP), nephropathy, cardiovascular complications, pneumonitis, alveolar hemorrhage, meningitis, and hepatitis [8]. In phase two and three clinical trials, alemtuzumab has established greater efficacy within 24-36 months than subcutaneous interferon beta-1a (SC IFNb-1a; Rebiff) used three times per week [9-11]. It has also decreased the volume of brain loss and lesion activity on magnetic resonance imaging (MRI) [10,11]. In a comparison of alemtuzumab with Rebiff in phase two clinical trial (CAMMS223) and phase three trial (CARE-MS), phase two trial showed fewer disability outcomes with alemtuzumab [9-11].

Due to safety issues and secondary autoimmune diseases associated with alemtuzumab, concerns were raised as the European Medical Association (EMA) decided to approve it as a first-line drug for relapsing-remitting MS (RRMS). However, the Food and Drug Association (FDA) did not support it, although these autoimmune adverse effects are rare and can be treated if they are diagnosed early [12]. This article's objective is to review whether alemtuzumab can be categorized as a first-line intervention for MS despite its adverse effects by comparing its efficacy and cost-effectiveness with other drugs introduced in the healthcare market.

## Review

Phenotypic classification of MS

Based on the phenotypical expression of disease, MS is divided into clinically isolated syndrome (CIS), RRMS, secondary progressive MS (SPMS), primary progressive MS (PPMS). CIS expresses initially in 80% of patients as an acute inflammation of various central nervous system (CNS) sites. After 20 years of duration, the disease expression progresses in 21% of patients to RRMS depending on clinically silent white matter lesions in MRI [13,14]. These early stages are manifested by the migration of autoreactive T and B cells across the blood-brain barrier, causing microglial cell activation, oxidative damage, mitochondrial injury, and neuronal cells' demyelination to develop distinguished plaques [15,16]. Based on the evidence of finding new T2 or gadolinium-enhanced lesions on MRI and clinical outcomes in over a year, these subtypes of MS can be further classified into active and inactive forms. MS affects the young adult population commonly, and the female-to-male ratio of occurrence of the disease is 2:1.

Effectiveness of alemtuzumab as compared to other disease-modifying therapies (DMTs)

Alemtuzumab has been approved for the treatment of RRMS with a favorable risk-benefit profile and long-term efficacy without the need for continuous administration. Since the complex pathogenesis of MS involves both inflammatory and neurodegenerative processes, it is imperative to closely monitor disease progression as it can lead to chronic disability. If alemtuzumab is used for RRMS, it improves disability and disease relapse rates. So, alemtuzumab is preferably used for patients in the relapsed stage. We will briefly compare the efficacy of alemtuzumab with other drugs approved for the treatment of MS.

Comparison of Alemtuzumab With Interferon-B

In 1993, subcutaneous interferon-b 1a was approved as a first-line treatment for RRMS [17]. Its action mechanism is to induce regulatory mediators’ production as interleukin IL-10 and IL-4, inhibiting proinflammatory cytokines (IFN-gamma, IL-17, TNF-a, and osteopontin) and blocking cell trafficking across the blood-brain barrier [18]. In a comparison of alemtuzumab with Rebiff (CARE-MS I) and (CARE-MS II), clinical trial patients with active disease who were enrolled in the study were taking subcutaneous interferon-b (SC IFN-b) for two years and had an inadequate response to this drug. The efficacy and sustainability of this medication were compared to alemtuzumab. After switching to this drug, our data showed that after five years of follow-up, cerebral atrophy and disease activity were reduced, and there were improvements in expanded disability status score (EDSS) and annualized relapse rate (ARR). There were marked improvements in disability, and 43% of patients developed confirmed disability improvement (CDI). Most patients were free of confirmed disability worsening (CDW). In most patients, there were no active lesions in the MRI and achieved no evidence of disease activity (NEDA) through extension (3-5 years) [10,11].

Comparison With Glatiramer Acetate and Dimethyl Fumarate

Glatiramer acetate (GA) was approved in 1995 to treat RRMS and was sold in the market as Copaxone. Its mechanism of action is to shift immune response driven by T-helper 1 cells to T-helper 2 cells through interaction with CD4 and CD8 cells and antigen-presenting cells (APC) [19]. Dimethyl fumarate (DMF) was approved in 2013 as a first-line oral drug for RRMS by FDA and EMA at the same time. Its mechanism of action is believed to be cytokine modulation and neuroprotective effects mediated by elongation-factor (EF-2). In two sizable controlled phase three trials, it was proved that DMF is superior to GA in controlling symptoms and improving EDSS. A prospective cohort observational study showed that GA reduces relapse and EDSS but works slower [20].

Comparison of Alemtuzumab With Fingolimod

Fingolimod was the first-line oral drug approved by the FDA in 2010 and by EMA in 2011 as the second line for RRMS. Its mechanism of action is to block the transport of lymphocytes out of the lymphatic tissue by binding to sphingosine 1 phosphate (SIP-1) receptors on the lymphocytes and preventing an invasion of brain tissue [21,22]. Freedoms and Transforms study demonstrated that fingolimod showed favorable efficacy than IFN-b, teriflunomide, and GA [23]. However, prospective studies that can compare fingolimod with alemtuzumab and natalizumab are lacking. Few placebo-controlled studies and MS-BASE registry findings suggest that fingolimod is less productive than alemtuzumab and natalizumab [24].

Comparison With Natalizumab

Natalizumab, a monoclonal antibody with an associated mechanism of action as an antagonist of α4β1-integrin, inhibits inflammatory cell migration across the blood-brain barrier [25]. Polman et al. showed that patients treated with natalizumab had a reduced disability rate evaluated by EDSS as 48% compared to placebo after two years [26]. Clinical relapse had decreased by 68%, with minimization of MRI lesions by 92%. On the other hand, Farrell and Geovanoni showed that there was a reduction in relapse when patients were treated with alemtuzumab, but EDSS score and MRI lesions were increased in MS patients (Table 1). Based on the data obtained from this study, it was proved that natalizumab has better efficacy than alemtuzumab. Furthermore, the current study showed that adverse reactions were mild when patients were treated with natalizumab than alemtuzumab, as there were less intense ITP, Graves' disease, nausea, and respiratory tract infection symptoms [27]. Here is a table summarizing a comparison of alemtuzumab with other disease-modifying therapies.

**Table 1:** Comparison of alemtuzumab with other DMTs CARE-MS: comparison of alemtuzumab with Rebiff efficacy in multiple sclerosis, NEDA: no evidence of disease activity, EDSS: expanded disability status score, CDI: confirmed disability improvement, SIP-1: sphingosine 1 phosphate receptors, IFN-B: interferon-beta, EF-2: elongation factor-2, IL-17: interleukin-17, IFN-gamma: interferon-gamma, TNF-a: tumor necrosis factor-a, TH-1: T-helper 1.

Disease-modifying therapies	Mechanism of action	Effects on expanded disability status score (EDSS)
Alemtuzumab	Depletion of B and T cells via CD-52 ligand	Most patients showed no evidence of disease activity (NEDA) after three-five years [10,11].
IFN-B	Induce production of regulatory cytokines (IL-10 and IL-4) and inhibiting the production of inflammatory cytokines (IL-17, IFN-gamma, TNF-a, osteopenin)	CARE-MS I, CARE-MS II, and CAMSS223 showed marked improvement in the EDSS score as 43% of patients showed confirmed disability improvement (CDI) when switched from IFN-b to alemtuzumab [10,11].
Glatiramer acetate	Shifting of the immune response from TH-1 cells to TH-2 cells	Glatiramer acetate improved EDSS but at a slower rate as compared to alemtuzumab [20].
Fingolimod	Block transport of lymphocytes out of the lymphatic tissue by binding to SIP-1 receptors on lymphocytes	Few placebo-controlled studies showed that fingolimod is less productive than alemtuzumab in reducing EDSS [24].
Natalizumab	The antagonist of α4β1-integrin inhibits inflammatory cells' migration across the blood-brain barrier.	Polman et al, Farrel, and Geovanni showed that natalizumab is more productive than alemtuzumab in reducing EDSS [26,27].
Dimethyl fumarate	Cytokine modulation and neuroprotective effects mediated by EF-2	Dimethyl fumarate reduces EDSS but at a slower rate [28].

Adverse effects caused by alemtuzumab and their monitoring

The most common adverse reactions encountered in patients treated with alemtuzumab were autoimmune thyroid disease, ITP, immune-mediated nephropathies, infusion reactions, and infections. Other rare effects were neutropenia, granulocytopenia, and pancytopenia.

Immune-Mediated Thrombocytopenic Purpura

Another autoimmune disease caused by alemtuzumab is immune-mediated thrombocytopenia (ITP). CAMMS223 study was stopped when three patients had ITP, and one patient died of intracerebral hemorrhage before he was diagnosed. Later, three more patients were diagnosed with ITP in phase three and open-label cohorts, and the rate was one percent and three percent, respectively [29,30]. ITP tends to present late in MS patients treated with alemtuzumab and is responsive to treatment with corticosteroids, intravenous IgG, platelet transfusion, and rituximab. Patients treated with alemtuzumab should get a complete blood count with differential done at monthly intervals for 48 months after the last infusion. After 48 months, tests should be done based on clinical findings, and a complete blood count should be obtained if immune-mediated thrombocytopenia is suspected in a patient. 

Autoimmune Thyroid Disease

In alemtuzumab-treated patients, the course of Graves' disease is indolent, and most thyroid eye disease (TED) cases were mild [31]. Some of the data confirmed a full spectrum of symptoms ranging from mild to severe, refractory to the most potent regimen. The disease has an unpredictable course exhibiting a high frequency of thyrotropin receptor antibodies (TRAB) positive hypothyroidism symptoms following the hyperthyroid illness. The phenomenon behind this autoimmunity is the proliferation of T cells, CD4+ and CD8+ cells, B cells survival, and class switching, thus causing inflammation through the action of IL-21 [32]. The disease period is also crucial as CD4+ took 35 months, and B cells took seven months to recover and then rise to 124% baseline post-treatment [33,34]. The disease time course guides us to monitor and manage the illness as six of our patients exhibit Graves' disease from six months to seven years. Still, the peak of symptoms is around 18-36 months [35,36]. So, it was concluded that patients treated with alemtuzumab should be monitored and reviewed for TRAB levels from six months to five years post-treatment [37]. And patients with high TRAB levels should get ophthalmologist evaluation earlier and before radioiodine treatment as it is a known risk factor for TEDs [38,39].

*Alemtuzumab-Associated Nephropathy* 

Patients treated with alemtuzumab developed immune-mediated renal adverse effects. In 2006, MS patients had glomerular basement membrane antibodies (anti-GBM) disease, following treatment with alemtuzumab, which was life-threatening and needed immediate follow-up and therapy [40]. It was reported in post-marketing surveillance that monthly blood and urine analysis should be done until 48 months after treatment with alemtuzumab. Membranous nephropathy is not a clinical emergency and requires only symptomatic treatment, but anti-GBM disease requires immediate treatment with cyclophosphamide, corticosteroids, and plasmapheresis.

Opportunistic Infections Associated With Alemtuzumab

The main biological effect observed during the treatment with alemtuzumab is lymphopenia as opportunistic infection rates increase after treatment. Almost 60%-70% of patients receiving 12 mg of alemtuzumab developed mild-to-moderate infections (vs. 45%-66% of patients receiving interferon-b 1a injections), as was evidenced by CARE-MS studies. The most commonly encountered diseases include upper respiratory, herpes simplex, zoster virus, and urinary tract infections [29,30]. Some other conditions were observed in open-label studies, including listeria meningitis, pyogenic granuloma, and gingivitis. In CAMMS223, one more case of listeria meningitis was encountered, and two more cases were recently observed [9].

Alemtuzumab-Associated Malignancy

The incidence of malignancy in patients treated with alemtuzumab has not been very high. Clinical trials mostly showed lymphoproliferative malignancies such as lymphoma, Castleman disease, and non-Epstein-Barr virus lymphoma. In CAMMS223, the most commonly encountered cancers after the treatment with alemtuzumab include breast cancer, non-Epstein-Barr virus lymphoma, and cervical cancer after 22-64 months of the therapeutic course [10]. There are much less data available regarding the risk of cancer in a patient treated with alemtuzumab. In the CARE-MS I study, the cancer rate was 0.5% vs. 0% in patients treated with 12 mg of alemtuzumab vs. interferon-b 1a group [11]. In the CARE-MS II study, 0.6% of patients developed cancer on being treated with 12 mg of alemtuzumab vs. 1.5% on being treated with interferon-b 1a [41].

Alemtuzumab Effects on Pregnancy

Alemtuzumab's effects on pregnancy are still not clear as data is quite limited. Alemtuzumab can be a risk to the growing fetus as this drug can cross the placenta. Newborns might get Graves' disease as TRAB can cross the placenta, and the mother can transiently transfer the disease to the fetus [42]. So, it is recommended that alemtuzumab is contraindicated for mothers of childbearing age for at least up to four months [43]. Achiron et al. demonstrated that no adverse effects were observed in the clinical development program in patients taking alemtuzumab. It was reported that, in the clinical trial, out of 1,486 patients (64.8% female), 179 pregnancies occurred in 131 patients. In completed pregnancies, 104 patients (66%) had live births, 36 patients (21%) had spontaneous abortions, 16 patients (9%) had elective abortions, and 1 patient (0.6%) had stillbirth. Infants who were delivered showed no congenital abnormalities, and rates of spontaneous abortion reported were similar to those in the general population [10].

Infusion-Associated Reactions of Alemtuzumab

**Figure 1:**
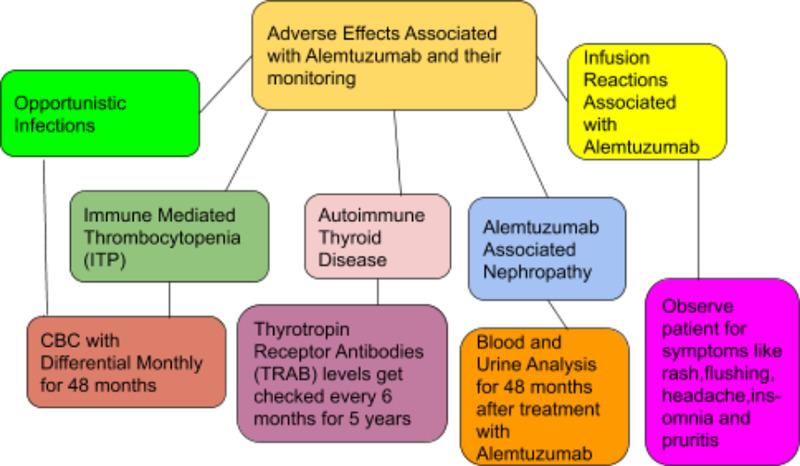
Adverse effects associated with alemtuzumab and their monitoring CBC: complete blood count, TRAB: thyrotropin receptor antibodies, ITP: immune-mediated thrombocytopenia.

Infusion-associated reactions (IAR) were observed in >90% of patients who took alemtuzumab in phase three clinical trial, and responses were mild to moderate in severity, including symptoms of rash, headache, fever, nausea, flushing insomnia, and pruritus (Figure 1) [44]. It was also reported that 10% of patients showed cardiac disorders, including tachycardia. Therefore, a physician must be aware of these side effects to monitor and treat the patient [45]. Pre-treatment with corticosteroids, antipyretics, and antihistamines can mitigate the IAR. Figure 1 depicts the adverse effects associated with alemtuzumab treatment and their monitoring.

Cost-effectiveness of alemtuzumab

As compared to other DMTs that need to be administered on a daily dose, the administration of alemtuzumab is different in a way that it has a long duration of effects and is given in two annual doses, administered intravenously. The first treatment course is offered in five consecutive days, and the second treatment is given in three consecutive days after 12 months. Alemtuzumab is the most cost-effective drug treatment for RRMS compared with other DMTs because it provided durable efficacy in the absence of the continuous need to administer it for patients.

Limitations

1. We did not have full access to the clinical data in the context of practical recommendations for alemtuzumab. We need to repeat treatment with the recrudescence of disease activity after many annual treatment cycles.

2. We cannot determine how many patients are being treated with alemtuzumab as it is not in the public domain, so it is difficult to conclude the treatment's frequency of adverse outcomes.

3. We might have missed cases that are not reported to PubMed.

## Conclusions

In recent years, treatment options have markedly increased in the context of treatment of RRMS, as dozens of different preparations are available in the market, having varied modes of transmission, safety profiles, efficacy, and mechanism of actions creating a complex network for physicians. Alemtuzumab has diverse effects on MS patients including positive and negative autoimmune adverse effects though it remains unclear whether it can be considered as a first-line option as compared to other DMTs. Despite its high-risk safety profile, alemtuzumab still offers a commendable therapeutic option because of its cost-effectiveness, unique dosing regimen, and long durability of action but not the first-line option. Effective monitoring of adverse effects and patient education might help initiate early management and identification of autoimmune diseases associated with alemtuzumab treatment.
